# Downregulation of Endogenous Hydrogen Sulfide Pathway Is Involved in Mitochondrion-Related Endothelial Cell Apoptosis Induced by High Salt

**DOI:** 10.1155/2015/754670

**Published:** 2015-05-11

**Authors:** Yanfang Zong, Yaqian Huang, Siyao Chen, Mingzhu Zhu, Qinghua Chen, Shasha Feng, Yan Sun, Qingyou Zhang, Chaoshu Tang, Junbao Du, Hongfang Jin

**Affiliations:** ^1^Department of Pediatrics, Peking University First Hospital, Beijing 100034, China; ^2^Key Laboratory of Molecular Cardiology, Ministry of Education, Beijing 100191, China; ^3^Department of Physiology and Pathophysiology, Peking University Health Science Centre, Beijing 100191, China

## Abstract

*Background*. The study aimed to investigate whether endogenous H_2_S pathway was involved in high-salt-stimulated mitochondria-related vascular endothelial cell (VEC) apoptosis. *Methods*. Cultured human umbilical vein endothelial cells (HUVECs) were used in the study. H_2_S content in the supernatant was detected. Western blot was used to detect expression of cystathionine gamma-lyase (CSE), cleaved-caspase-3, and mitochondrial and cytosolic cytochrome c (cytc). Fluorescent probes were used to quantitatively detect superoxide anion generation and measure the *in situ* superoxide anion generation in HUVEC. Mitochondrial membrane pore opening, mitochondrial membrane potential, and caspase-9 activities were measured. The cell apoptosis was detected by cell death ELISA and TdT-mediated dUTP nick end labeling (TUNEL) methods. *Results*. High-salt treatment downregulated the endogenous VEC H_2_S/CSE pathway, in association with increased generation of oxygen free radicals, decreased mitochondrial membrane potential, enhanced the opening of mitochondrial membrane permeability transition pore and leakage of mitochondrial cytc, activated cytoplasmic caspase-9 and caspase-3 and subsequently induced VEC apoptosis. However, supplementation of H_2_S donor markedly inhibited VEC oxidative stress and mitochondria-related VEC apoptosis induced by high salt. *Conclusion*. H_2_S/CSE pathway is an important endogenous defensive system in endothelial cells antagonizing high-salt insult. The protective mechanisms for VEC damage might involve inhibiting oxidative stress and protecting mitochondrial injury.

## 1. Introduction

High-salt diet as an important risk factor of hypertension could interrupt body homeostasis, resulting in cardiovascular diseases and even life-threatening events. High-salt insult can promote the proliferation of vascular smooth muscle cells induced by angiotensin II, significantly increase blood pressure in salt-sensitive rats, and accelerate hypertensive process and the development of cardiovascular disease. Moreover, it can change small artery structures in normal-diet rats.

Numerous studies have been conducted to investigate the mechanisms for hypertension. Previous studies indicated that high-salt insult promoted hypertension in association with activating angiotensin-aldosterone system [1], damaging renal natriuresis function [2], enhancing sympathetic activity [3], increasing the extracellular capacity, and eventually leading to sodium and water retention [4]. In addition, high salt could destroy the transfer passage of paraventricular nucleus G*α*i-protein gating signals [5]. However, the mechanisms for high-salt-induced hypertension remain unclear. Vascular endothelial cell serves as a junction of blood flow and vessel wall. Maintenance of vascular endothelium integrity is essential for smooth blood flow, which plays an important role in maintaining vascular homeostasis [6]. Endothelial dysfunction is the initial cause of the onset and development of a variety of cardiovascular diseases [7]. Importantly, vascular endothelium dysfunction plays a key role in the pathogenesis of salt-sensitive hypertension, while high-salt stimulation could damage vascular endothelial function [8]. High salt led to endothelial cell cortex sclerosis via compromising epithelial sodium channels [9] and contributing to intracellular edema and mitochondrion swelling [10] while the mechanisms for intracellular structural changes and endothelial dysfunction are unidentified. In normotensive rats, high-salt diet increased production of reactive oxygen species in striated muscle and superior mesenteric arteries, activating the oxidative stress system [11, 12]. Therefore, we speculated that high-salt-induced oxidative damage might be involved in the development of cell damage induced by high salt. H_2_S is known as a gas with rotten egg stink and toxicity. Recent studies suggest that endogenous H_2_S exerts biological effects and acts as a novel gaseous signal molecule [13–19]. Several studies indicated that H_2_S/CSE pathway was downregulated in the development of hypertension, while H_2_S supplementation could significantly lower blood pressure and reverse aortic remodeling [20, 21]. It was also implied that H_2_S could protect human umbilical vein endothelial cells (HUVECs) via antagonizing oxidative stress-related pathways [22]. Hence, we proposed a hypothesis that downregulation of endogenous H_2_S/CSE might be involved in mitochondrion-related endothelial cell injury induced by high-salt exposure.

Therefore, the present study was undertaken to explore the possible effects of endogenous H_2_S on endothelial apoptosis under high-salt stimulation and its mechanisms.

## 2. Materials and Methods

### 2.1. Cell Culture

HUVECs were purchased from Lifeline Cell Technology, USA. The 3–7 generations of HUVEC were used in this experiment. HUVECs were cultured in the medium which was supplemented with 0.2% FBS, 0.1% rh VEGF, 0.1% rh IGF-1, 0.1% rh FGF-b, 0.1% ascorbic acid, 0.1% rh EGF, 0.1% heparin, 0.1% Hydrocort, and 5% L-glutamine (Lifeline Cell Technology, USA) in an incubator containing 5% CO_2_ at constant temperature of 37°C. The cells were cultured in exogenous growth factors-free medium and synchronized for 12 h before each experiment. The concentration of sodium in the synchronization medium was 137 ± 1.0 mmol/L. Sodium chloride (Sinopharm, Shanghai, China) was added to culture medium at different concentrations including 150 mmol/L, 200 mmol/L, and 250 mmol/L.

### 2.2. Measurement of Endogenous H_2_S Content in HUVEC

Endogenous H_2_S in cells was measured using a fluorescent probe (provided by Professor Xinjing Tang, Peking University Health Science Centre, China) as described previously [23]. The culture supernatant was collected to test H_2_S levels in HUVEC. Then, the slides were washed with PBS (0.01 mol/L) for three times and fixed in prewarmed 4% paraformaldehyde at room temperature for 20 min. After washing with PBS three times, the slides were stained in the working liquid of fluorescent probe for 30 min at 37°C. The slides were mounted by antifade solution (Applygen, Beijing, China) after washing with PBS. Then, the slides were detected as blue fluorescent by laser confocal scanning microscope. The concentration of H_2_S in culture supernatant was detected by the free radical analyzer TBR4100 (World Precision Instruments, USA). First, the ISO-H_2_S-100 sensor was polarized with PBS buffer solution (pH 7.2, 0.05 mol/L) to achieve a stable baseline current (usually between 100 and 2000 pA). Then, the sensor was calibrated by the standards, in which the concentration of H_2_S was 0.5 *μ*mol/L, 1 *μ*mol/L, 2 *μ*mol/L, 4 *μ*mol/L, 8 *μ*mol/L, and 16 *μ*mol/L, respectively. The current output jumped rapidly after each sample and then plateaus. As soon as it reached a plateau, the next sample was injected. The reduction difference between the peak and baseline was recorded (pA). Then, the calibration curve was constructed by plotting the reduction difference (pA) against the concentration (*μ*mol/L) of H_2_S. The sensor tip was immersed into each cultural supernatant sample in about 10–15 mm deep, and the concentration of H_2_S was calculated via the equation.

### 2.3. Western Blotting Analysis

Expressions of CSE, cytochrome c (cytc), and caspase-3 as well as cleaved-caspase-3 in HUVEC were detected by western blot. The total protein of HUVEC was extracted using ice-cold cell lysis buffer containing 50 mmol/L Tris-HCl, 150 mmol/L NaCl, 1 mmol/L PMSF, 1 mmol/L EDTA, 5 *μ*g/mL aprotinin, 5 *μ*g/mL leupeptin, 1% Triton X-100, 1% sodium deoxycholate, and 0.1% SDS. The cells were washed with ice-cold PBS for three times, soaked in ice-cold lysis buffer for 30 min, and scraped off. Cell lysate was subsequently centrifuged at 12000 g for 10 min at 4°C, and supernatant was reserved. Protein concentration was detected using BCA method. Equal amount of protein sample (60 *μ*g) was loaded, and SDS-PAGE electrophoresis of 10% or 12% separation gel was conducted. The separated proteins were transferred onto a nitrocellulose membrane for 2 h at 200 mA after electrophoresis. The membranes were blocked with 5% dried skimmed milk for 1 h. Then, the primary antibodies CSE (Sigma, USA) (dilution 1 : 200), cytc (Santa Cruz, USA) (dilution 1 : 200), caspase-3 (Cell Signaling Technology, USA) (dilution 1 : 1000), and cleaved-caspase-3 (Cell Signaling Technology, USA) (dilution 1 : 200) were added, respectively, and incubated at 4°C overnight. The second antibody was incubated for 1 h at room temperature after washing. LumiGLO chemiluminescence reagent was used and exposed to films. Quantification of blots was analyzed using an AlphaImager (San Leandro, CA, USA).

### 2.4. Preparation of Mitochondrial Protein

Separation of mitochondrion and cytoplasm was conducted using Mitochondria Isolation Kit (Applygen, Beijing, China). The HUVECs were mixed thoroughly with Mito solution after centrifugation at 800 g for 5 min, at 4°C. The supernatant was collected in a fresh tube on ice after centrifuging at 800 g for 5 min, at 4°C twice. Supernatant containing cytoplasm was then collected after centrifuging at 10000 g for 20 min, at 4°C, while precipitate containing mitochondria was processed for further centrifugation. The precipitate was washed with Mito solution and centrifugated at 12000 g for 10 min, at 4°C. The sedimentation was dissolved in Mito solution.

### 2.5. Detection of Superoxide Anion in HUVEC by Dihydroethidium (DHE) and CellROX Green Reagent

DHE (Beyotime, Shanghai, China) was used to detect superoxide anion in HUVEC. The medium covering the cells was changed after washing three times with PBS. Then, the cells were incubated with DHE probes for 30 min at 37°C, avoiding light exposure. Subsequently, the cells were mounted in new cultural medium for observation under a fluorescence microscope. Moreover, CellROX Green Reagent (Thermo Fisher, MA, USA) was used to quantify the generation of superoxide anion in HUVEC. HUVECs were plated in a 96-well plate. CellROX Green Reagent at a final concentration of 5 *μ*mol/L was added in the medium and incubated with cells at 37°C for 60 min. The cells were then washed with PBS and analyzed on Fluoroskan Ascent Fluorometer (Thermo Fisher, MA, USA). Fluorescence intensity was analyzed at the excitation/emission wavelengths of 488/520 nm.

### 2.6. Detection of Mitochondrial Superoxide Anion in HUVEC by MitoSOX Reagent

MitoSOX Red Mitochondrial Superoxide Indicator (Thermo Fisher, MA, USA) was used to detect the generation of mitochondrial superoxide anion in HUVEC and MitoTracker Green FM (Thermo Fisher, MA, USA) was used to label mitochondria. The HUVEC on slides were covered with MitoSOX (5 *μ*mol/L) and MitoTracker probes (100 nmol/L) after washing with PBS. The slides were incubated for 20 min at 37°C, avoiding light. Then, the HUVECs were fixed in prewarmed 4% paraformaldehyde at room temperature for 15 min after washing with warm PBS for three times. The slides were mounted by antifade solution (Applygen, Beijing, China) after washing with PBS. Cells on the slides were detected by a laser scanning confocal microscope. Red fluorescence indicated mitochondrial superoxide anion and green fluorescence indicated labeled mitochondria.

### 2.7. Detection of Mitochondrial Permeability Transition Pore (MPTP) Opening in HUVEC

Cell MPTP Assay Kit (Genmed, Shanghai, China) was used to test the MPTP opening in HUVEC. Experiment rationale was that calcein-AM could gather in mitochondrion presenting green fluorescent staining, whereas, being released into the cytoplasm via the opening of MPTP, fluorescent quenching could occur. Culture medium was discarded and slides were rinsed gently with cleaning solution. Slides were subsequently incubated with staining working solution for 30 min at 37°C, protecting from light. 4% paraformaldehyde was used to fix the cells for 15 min after rinsing for three times with cleaning solution. Finally, the slides were mounted with antifade solution (Applygen, Beijing, China) after washing with PBS three times and were immediately examined using a laser confocal microscope.

### 2.8. Measurement of Mitochondrial Membrane Potential in HUVEC

Mitochondrial membrane potential in HUVEC was measured by Mitochondrial Membrane Potential Assay Kit with JC-1 (Beyotime, Shanghai, China). When mitochondrial membrane potential is high in living cells, JC-1 accumulates in matrix in the form J-aggregates presenting red fluorescence; while mitochondrial membrane potential is low in apoptotic cells, JC-1 cannot aggregate, and the JC-1 monomer presents green fluorescence. Decreased mitochondrial membrane potential suggests apoptosis. Briefly, JC-1 working solution and cell medium were mixed at the ratio of 1 : 1. Then, the slides seeded with HUVEC were incubated with the mixture for 20 min at 37°C in the dark. The cells were fixed in prewarmed 4% paraformaldehyde at room temperature for 20 min after washing with ice-cold JC-1 buffer solution twice. After washing three times with PBS, the antifade solution (Applygen, Beijing, China) was used to mount the slides. Analysis was conducted immediately using a laser confocal scanning microscope.

### 2.9. *In Situ* and Quantitative Measurement of Caspase-9 Activity Assay in HUVEC

Change of* in situ* caspase-9 activity in HUVEC was detected by living cells caspase-9 activity fluorescence staining kit (Genmed, Shanghai, China). Briefly, after washing twice with PBS buffer, the cells on glass slide were incubated with fluorescence probe FITC-LEHD-FMK, a FITC labeling inhibitor of caspase-9, in room temperature for 30 min. 4% paraformaldehyde was used to fix the cells in room temperature for 30 min after washing three times with PBS buffer. Then the antifade solution was used to mount the slides. Observations were made immediately under a laser confocal scanning microscope.

Change of caspase-9 activity in HUVEC was quantified using cell caspase-9 colorimetric activity kit (Genmed, Shanghai, China). The assay was based on spectrophotometric detection of the chromophore p-nitroaniline (pNA) cleavage by caspase-9 from the labeled substrate Ac-LEHD-pNA. The free pNA was quantified using a microtiter plate reader at 405 nm. Briefly, after washing twice with PBS buffer, the cells were incubated with lysis buffer on ice for 30 min. The cells were subsequently scraped off carefully and centrifuged for 5 min at 16000 g in a microcentrifuge. The supernatant was collected in a fresh tube on ice. The BCA method was used for protein concentration determination. Then 50 *μ*g sample and substrate buffer were put into 96-well plate in order and incubated for 90 min at 37°C. Finally, samples were read in a microtiter plate reader (Bio-Rad, Hercules, CA, USA) at 405 nm.

### 2.10. *In Situ* and Quantitative Detection of Apoptosis in HUVEC by Using TdT-Mediated dUTP Nick End Labeling (TUNEL) Assay and ELISA Assay

*In situ* apoptosis of HUVEC was detected with* in situ* Cell Death Detection Kit, Fluorescein (Roche, Mannheim, Germany) in accordance with the instructions of the manufacturer. Briefly, the cells on slides were washed for three times with PBS. Then, the slides were covered with TUNEL reaction mixture and incubated for 60 min at 37°C in dark. The cells were fixed in prewarmed 4% paraformaldehyde at room temperature for 15 min after washing with PBS. Then, the antifade solution was used to mount the slides after washing three times with PBS, and the slides were immediately transferred for analysis under a laser confocal scanning microscope. Moreover, the quantitative detection of DNA fragments in HUVEC was measured by Cell Death Detection ELISA^PLUS^ Kit (Roche, Mannheim, Germany) according to the suggested protocol of the manufacturer. The assay is based on a quantitative sandwich-enzyme-immunoassay principle using mouse monoclonal antibodies directed against DNA and histones, respectively. This allows the specific determination of mono- and oligonucleosomes in the cytoplasmatic fraction of cell lysates. Briefly, the cell lysate was added into a streptavidin-coated microplate. A mixture of anti-histone-biotin and anti-DNA-POD was added and incubated. During the incubation period, the antibody-nucleosome complexes were bound to the microplate by the streptavidin. After a washing step, the unbound components were removed. The amount of nucleosomes by the POD retained in the immunocomplex was quantitatively determined by a microtiter plate reader (Bio-Rad, CA, USA) at 405 nm.

### 2.11. Statistical Analysis

Data are described as mean ± SE. SPSS 13 (SPSS Software, Inc., Chicago, IL, USA) was used for data analysis. Independent-samples *t*-test or one-way ANOVA followed by either LSD test was conducted and *P* < 0.05 was considered statistically significant.

## 3. Results

### 3.1. The Endogenous H_2_S Pathway Was Downregulated in High-Salt- (HS-) Stimulated HUVEC

**Figure 1:**
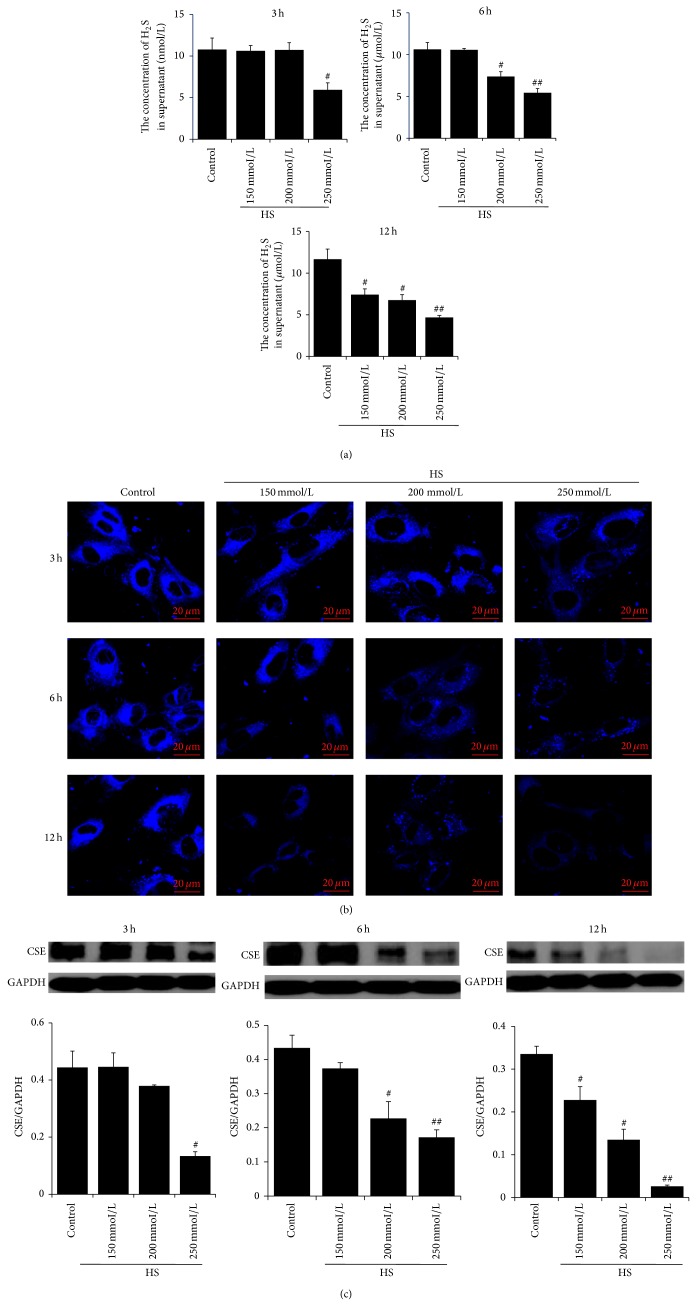
Changes in endogenous H_2_S pathway in human umbilical vein endothelial cells (HUVECs). (a) The concentration of endogenous H_2_S in supernatant detected by the free radical analyzer TBR4100; (b) the production of endogenous H_2_S in HUVEC determined by H_2_S fluorescent probe; and (c) CSE protein expression in HUVEC analyzed by western blotting. Control: the concentration of sodium was 137 mmol/L. HS: high salt; the concentration of sodium was 150 mmol/L, 200 mmol/L, and 250 mmol/L, respectively. The scale in (b) represented 20 *μ*m. ^##^ *P* < 0.01; ^#^ *P* < 0.05 versus control group.

Compared with control group, H_2_S synthesis in HUVEC was significantly reduced with prolonged sodium chloride (NaCl) incubation. After the treatment with 250 mmol/L NaCl medium for 3 h, H_2_S content in the culture supernatant of HUVEC was significantly decreased (Figure 1(a)), and the fluorescence of H_2_S probes weakened (Figure 1(b)). H_2_S content in the culture supernatant was significantly decreased after incubation with 200 mmol/L and 250 mmol/L NaCl medium for 6 h, respectively, with parallel decrease of the H_2_S probe fluorescence in HUVECs (Figure 1(b)). Moreover, 12 h incubation with 150 mmol/L, 200 mmol/L, and 250 mmol/L NaCl medium demonstrated a significantly decreased H_2_S content in HUVEC culture supernatant (Figure 1(a)) as well as reduced H_2_S probe fluorescence (Figure 1(b)). The results of CSE protein expression in HUVEC were consistent with the variation trend of H_2_S content (Figure 1(c)).

### 3.2. H_2_S Inhibited Superoxide Anion Generation in High-Salt-Induced HUVEC

**Figure 2:**
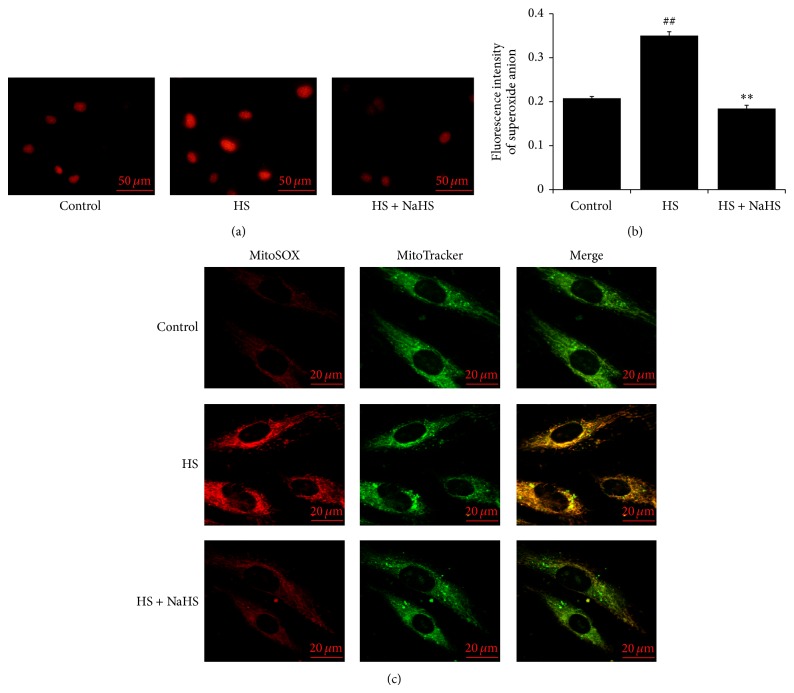
Changes in superoxide anion generation in human umbilical vein endothelial cells (HUVECs). (a) Fluorescent micrographs of superoxide anion generation in HUVEC detected by DHE probes. Red fluorescence indicates levels of superoxide anion generation in HUVEC; (b) quantification analysis of fluorescent intensity of superoxide anion generation in HUVEC detected by CellROX Green Reagent on Fluoroskan Ascent Fluorometer; (c) superoxide anion generation in HUVEC mitochondria detected by MitoSOX probes. Control: the cell treated with 137 mmol/L sodium. HS: high salt, the cell treated with 200 mmol/L sodium for 6 h. HS + NaHS: the cell pretreated with 200 *μ*mol/L NaHS for 30 min following 200 mmol/L sodium for 6 h. ^##^ *P* < 0.01 versus control group; ^∗∗^ *P* < 0.01 versus HS group.

Compared with control group, the superoxide anion generation in HUVEC of HS group represented by both DHE staining image and fluorescence quantification with CellROX Green Reagent on fluorometer was significantly increased (Figures 2(a) and 2(b)). However, when pretreated with NaHS, the superoxide anion generation in high-salt-induced HUVEC was decreased markedly (Figures 2(a) and 2(b)). Furthermore, the change of superoxide anion generation in mitochondria of HUVEC detected by MitoSOX probe was in accordance with the change of superoxide anion generation in whole HUVEC (Figure 2(c)).

### 3.3. H_2_S Inhibited Mitochondrial Dysfunction in High-Salt-Induced HUVEC

**Figure 3:**
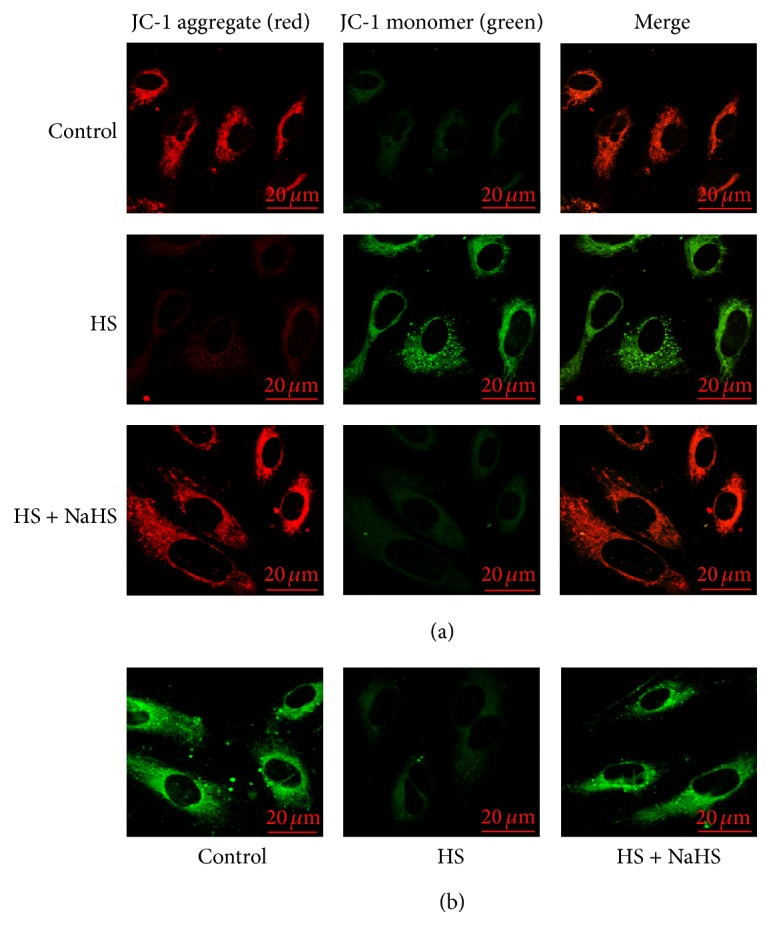
Changes in mitochondrial membrane potential and mitochondrial permeability transition pore (MPTP) opening in human umbilical vein endothelial cells (HUVECs). (a) Change of mitochondrial membrane potential detected by JC-1 fluorescent probe and examined by laser confocal microscope, with red fluorescence presenting JC-1 aggregate and green JC-1 monomer. (b) Changes of MPTP opening in HUVEC detected by calcein-AM as a fluorescence indicator by laser confocal microscopy. The green fluorescence quenching represented MPTP opening. Control: the cell treated with 137 mmol/L sodium. HS: high salt, the cell treated with 200 mmol/L sodium for 6 h. HS + NaHS: the cell pretreated with 200 *μ*mol/L NaHS for 30 min following 200 mmol/L sodium for 6 h.

In HS group, the mitochondrial membrane potential was significantly reduced (Figure 3(a)) and mitochondrial permeability transition pore significantly opened (Figure 3(b)) relative to that of the control group, whereas H_2_S donor increased mitochondrial membrane potential (Figure 3(a)) and closed mitochondria permeability transition pore in HS-treated HUVEC (Figure 3(b)).

### 3.4. H_2_S Antagonized High-Salt-Induced Release of Cytc from Mitochondria into Cytoplasm in HUVEC

**Figure 4:**
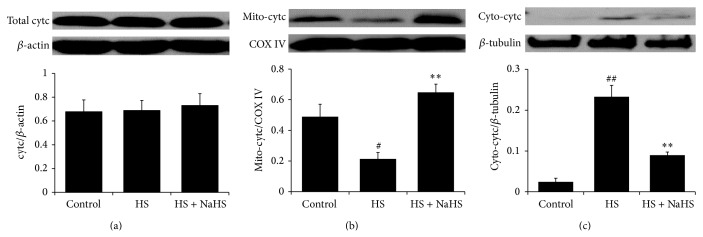
Cytochrome c (cytc) protein expression and distribution in human umbilical vein endothelial cells (HUVECs). Control: the cell treated with 137 mmol/L sodium. HS: high salt, the cell treated with 200 mmol/L sodium for 6 h. HS + NaHS: the cell pretreated with 200 *μ*mol/L NaHS for 30 min following 200 mmol/L sodium for 6 h. ^##^ *P* < 0.01, ^#^ *P* < 0.05 versus control group; ^∗∗^ *P* < 0.01 versus HS group.

Western blot results showed no significant difference of the total cytc among different groups. Interestingly, compared with control group, high-salt treatment significantly downregulated cytc protein expression in mitochondria of HUVEC (*P* < 0.05) and markedly increased cytoplasmic cytc (*P* < 0.01) (Figure 4), whereas, in the presence of NaHS, the cytoplasmic cytc protein expression was profoundly downregulated (*P* < 0.01) and mitochondria cytc was upregulated (*P* < 0.01) (Figure 4) in HS + NaHS-treated HUVEC as compared with that of HS-treated HUVEC.

### 3.5. H_2_S Inhibited Caspase-9 Activation in High-Salt-Treated HUVEC

**Figure 5:**
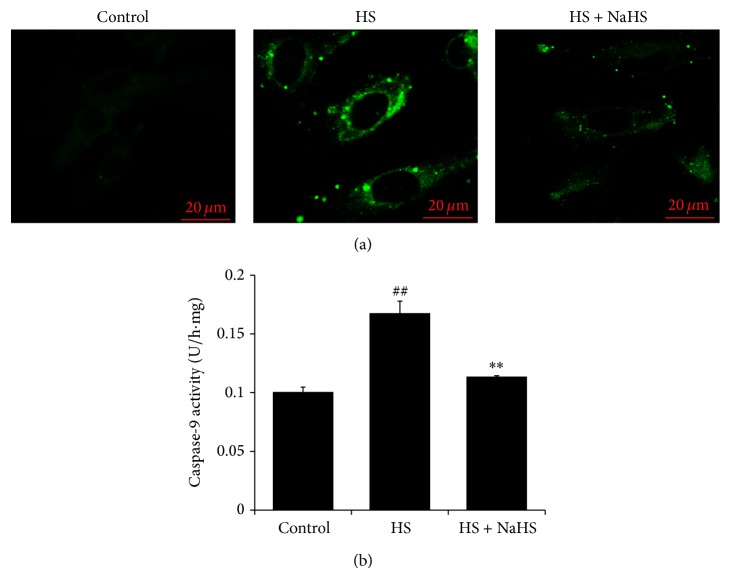
Caspase-9 activities in human umbilical vein endothelial cells (HUVECs). (a) Caspase-9 activities in HUVEC detected by living cells caspase-9 fluorescence staining kit. (b) Quantitative analysis of caspase-9 activities in HUVEC. Control: the cell treated with 137 mmol/L sodium. HS: high salt, the cell treated with 200 mmol/L sodium for 6 h. HS + NaHS: the cell pretreated with 200 *μ*mol/L NaHS for 30 min following 200 mmol/L sodium for 6 h. ^##^ *P* < 0.01 versus control group; ^∗∗^ *P* < 0.01 versus HS group.

Compared with the control group, incubation with high salt for 6 h markedly enhanced the green fluorescence indicating caspase-9 activities in HUVEC and semiquantitative analysis suggested profoundly increased caspase-9 activity (Figure 5), while H_2_S administration reversed the high-salt-induced caspase-9 activities (Figure 5).

### 3.6. H_2_S Reversed HS-Induced HUVEC Apoptosis

**Figure 6:**
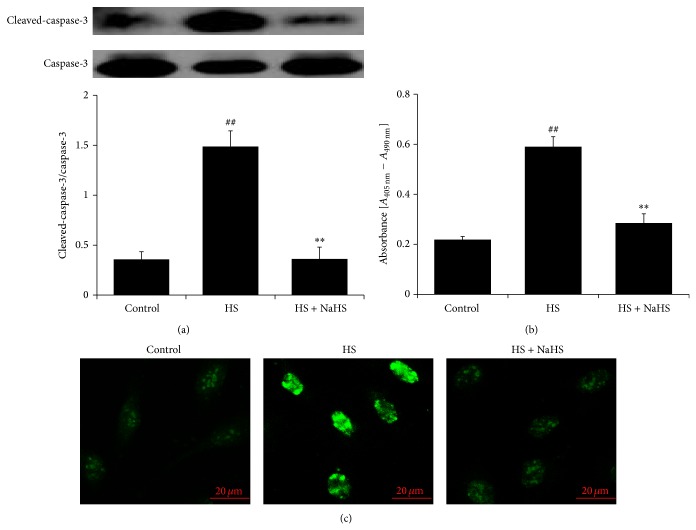
Apoptosis and caspase-3 activities in human umbilical vein endothelial cells (HUVECs). (a) Caspase-3 activities in HUVEC detected by western blot; (b) DNA fragmentation in HUVEC detected by Cell Death Detection ELISA. (c) HUVEC apoptosis detected by* in situ* Cell Death Detection Kit, Fluorescein. Control: the cell treated with 137 mmol/L sodium. HS: high salt, the cell treated with 200 mmol/L sodium for 6 h. HS + NaHS: the cell pretreated with 200 *μ*mol/L NaHS for 30 min following 200 mmol/L sodium for 6 h. ^##^ *P* < 0.01 versus control group; ^∗∗^ *P* < 0.01 versus HS group.

After incubation with high salt (200 mmol/L) for 6 h, the ratio of cleaved-caspase-3/caspase-3 in HUVECs was markedly increased (*P* < 0.01) (Figure 6(a)), the amount of nucleosomes in the HUVEC lysate representing DNA fragmentation was increased (*P* < 0.01) (Figure 6(b)), and the green fluorescence intensity in the nuclei of TUNEL-positive HUVECs was augmented (Figure 6(c)). Interestingly, H_2_S donor inhibited caspase-3 activation, and DNA fragmentation in HUVECs was treated with high salt (*P* all <0.01) (Figures 6(a) and 6(b)) and weakened the green fluorescence intensity in the nuclei of TUNEL-positive HUVECs (Figure 6(c)).

## 4. Discussion

High-salt diet is an important risk factor associated with hypertension. Recently, several studies have focused on investigating the direct impact of high salt on blood vessels. Liu et al. [24] found that high salt could promote the proliferation of vascular smooth muscle cells induced by angiotensin II. A recent study by Dmitrieva and Burg [25] proved that high salt might increase the secretion of von Willebrand factor (vWF) in vascular endothelial cells, resulting in hypercoagulability and thrombosis. Vascular endothelial cells serve as the primary barrier maintaining vascular function and structural stability. In the present study, we found that increased concentration of extracellular NaCl promoted caspase-3 activities and apoptosis in endothelial cells as demonstrated by TUNEL assay, confirming that high salt could stimulate endothelial cell apoptosis. Then, subsequently, the possible mechanism involved was examined.

The cardiovascular protective effects of endogenous H_2_S have been widely reported. Yang et al. [20] discovered that H_2_S generated by vascular endothelial cells played an important regulatory role in maintaining vascular structure and function and CSE deficiency was involved in the abolished endothelium-dependent vasodilation and hypertention in mutant mice. Shen et al. [22] reported H_2_S encouraged the proliferation and migration of endothelial cells and played a protective role in vascular endothelial cells. In our present study, H_2_S content in both vascular endothelia and culture supernatant showed a time-dependent reduction after high-salt treatment in addition to downregulated expression of CSE, a key enzyme for H_2_S synthesis. Nevertheless, supplementing H_2_S donor markedly inhibited endothelial cell apoptosis induced by high salt, suggesting that high-salt-induced downregulation of endogenous H_2_S pathway resulted in inadequate protective effects of H_2_S on vascular endothelial cells, which might be one of the mechanisms associated with high-salt-mediated vascular endothelial damage. Then, the possible protective mechanism by which endogenous H_2_S protected against high-salt damage in vascular endothelia was investigated.

Animal experiments revealed that high-salt diet increased production of microvascular reactive oxygen species in rat striated muscles as well as promoted superoxide anion generation in the superior mesenteric arteries, contributing to oxidative stress* in vivo* [11, 12]. Other studies established antioxidant effects of H_2_S in multiple systems of the body including the central nervous system, cardiovascular system, and respiratory system [19, 22, 26]. In the present study, DHE fluorescent probe demonstrated that high-salt stimulation significantly augmented superoxide anion generation in HUVEC. Considering that mitochondrion was one of major sources producing oxygen free radicals, in particular, increased production and decreased removal of oxygen free radicals caused by abnormal mitochondrial structures and dysfunctional mitochondria remain an important mechanism of cellular oxidative stress injury. Therefore, further study was designed to examine oxygen free radical generation in mitochondria using mitochondrial oxygen radical-specific fluorescent probe MitoSOX. The results showed that high salt promoted oxygen free radical generation in mitochondria, which was abolished in the presence of H_2_S. Therefore, we speculate that mitochondria play a crucial role in high-salt-induced vascular endothelial injury and likely mediate protective effects of endogenous H_2_S on vascular endothelia.

Mitochondria-related apoptosis is one of the important mechanisms for apoptosis [27]. Previous studies showed that excessive generation of mitochondrial ROS might cause lipid peroxidation of mitochondrial membranes, thereby destroying mitochondrial membrane potential and promoting mitochondrial MPTP opening; meanwhile, the combination of cytc and endometrial center phospholipid molecules was broken, releasing the “free” cytc. Increased mitochondrial permeability encouraged the leakage of cytc and other proapoptotic molecules into the cytoplasm, activating caspase-9 and caspase-3, initiating apoptosis cascade, and eventually culminating to cell death [28]. Our results showed that, under high-salt treatment, mitochondrial membrane potential in vascular endothelia was significantly reduced along with increased MPTP opening and release of mitochondrial cytc to cytoplasm, activating cytoplasmic caspase-9. In contrast, H_2_S donor restored mitochondrial membrane potential in vascular endothelia, inhibited MPTP opening, and blocked the leakage of mitochondrial cytc, establishing that H_2_S antagonized the mitochondria-mediated apoptosis induced by high salt.

Taken together, this study demonstrated for the first time that high salt damaged vascular endothelial cells through downregulating H_2_S/CSE pathway, which might subsequently result in augmented endothelial cell oxidative stress, increasing mitochondria-mediated endothelial cell apoptosis.

The limitation of this study was the relatively high dose of sodium used in the experiment. Although recent study showed that HUVECs adapted sodium well at 380 mosmol/kg (or 190 mmol/L sodium), maintaining a normal appearance of the cells and a logarithmic growth for two weeks [25], and the concentration of 500 mosmol/kg (or 250 mmol/L sodium) was used in the* in vitro* experiment [29], there was a limited elevation of plasma sodium concentration (about 2–6 mmol/L) in salt-sensitive hypertensive patients and animal models with high-salt diet [30, 31]. Moreover, extreme hypernatraemia (196 mmol/L) only occurred in a small part of patients [32]. Furthermore, sodium concentration at 150 mmoL and above is very likely to affect the membrane potential of various cells and tertiary protein structure of multiple enzymes and accordingly the enzyme activity, which might interrupt the specificity of the protection of endogenous H_2_S/CSE pathway on the HUVECs. Therefore, the effect of a reasonable high-salt insult which was in accordance with clinical change in plasma sodium in the patients and animal model with high-salt diet on the HUVEC needs further investigation.
